# Next-generation sequencing to guide cancer therapy

**DOI:** 10.1186/s13073-015-0203-x

**Published:** 2015-07-29

**Authors:** Jeffrey Gagan, Eliezer M. Van Allen

**Affiliations:** Department of Pathology, Brigham and Women’s Hospital, Boston, MA 02115 USA; Department of Medical Oncology, Dana-Farber Cancer Institute, Boston, MA 02115 USA; Broad Institute of MIT and Harvard, Cambridge, MA 02142 USA

## Abstract

As a result of multiple technological and practical advances, high-throughput sequencing, known more commonly as “next-generation” sequencing (NGS), can now be incorporated into standard clinical practice. Whereas early protocols relied on samples that were harvested outside of typical clinical pathology workflows, standard formalin-fixed, paraffin-embedded specimens can more regularly be used as starting materials for NGS. Furthermore, protocols for the analysis and interpretation of NGS data, as well as knowledge bases, are being amassed, allowing clinicians to act more easily on genomic information at the point of care for patients. In parallel, new therapies that target somatically mutated genes identified through clinical NGS are gaining US Food and Drug Administration (FDA) approval, and novel clinical trial designs are emerging in which genetic identifiers are given equal weight to histology. For clinical oncology providers, understanding the potential and the limitations of DNA sequencing will be crucial for providing genomically driven care in this era of precision medicine.

## Introduction

Many biological discoveries about cancer have been the product of a reductionist approach, which focuses on modeling phenomena with as few major actors and interactions as possible [1, 2]. This reductionist thinking led the initial theories on carcinogenesis to be centered on how many “hits” or genetic mutations were necessary for a tumor to develop. It was assumed that each type of cancer would progress through a similar, if not identical, process of genetic hits. Indeed, there are a handful of cancer types, such as chronic myelogenous leukemia, that feature a single and pathognomonic DNA mutation. Working on this assumption, early methods to explore the genomic foundations of different cancers involved targeted exploration of specific variants and genes in a low-throughput fashion [3]. However, most cancers are genetically complex, and are better defined by the activation of signaling pathways rather than a defined set of mutations. The success of the Human Genome Project inspired similar projects looking at the genome in various cancers [4]. That success, along with the increased affordability and reliability of sequencing [5], has led to the integration of genome science into clinical practice. The use of these data to assist in diagnosis is generally referred to as precision medicine [6, 7].

Next-generation sequencing (NGS), also known as massively parallel sequencing, represents an effective way to capture a large amount of genomic information about a cancer. Most NGS technologies revolve around sequencing by synthesis [5]. Each DNA fragment to be sequenced is bound to an array, and then DNA polymerase adds labeled nucleotides sequentially. A high-resolution camera captures the signal from each nucleotide becoming integrated and notes the spatial coordinates and time. The sequence at each spot can then be inferred by a computer program to generate a contiguous DNA sequence, referred to as a read.

**Fig. 1:**
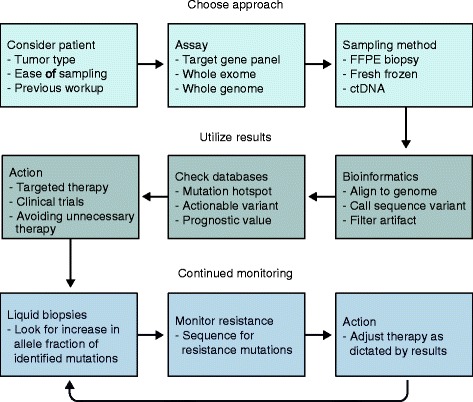
A summary of the workflow for NGS sequencing in oncology. The first row outlines selecting the appropriate sample and assay. Turning raw data into clinically actionable information is covered in the second row. The third row looks at how NGS may be used in the continued monitoring of disease. *ctDNA* circulating tumor DNA, *FFPE* formalin-fixed, paraffin-embedded specimen

Multiple technological enhancements have allowed NGS to be more readily implemented in a clinical workflow (Fig. 1). Samples now no longer need to be handled differently from standard diagnostic specimens, and recent advances have even enabled increasingly complex genomic data to be derived from a patient’s peripheral blood. The concept of precision medicine goes hand in hand with an understanding of the cancer genome as determined by NGS. In this review, we will explore the expanding NGS methodologies, analytical methods, and clinical applications that are driving precision cancer medicine.

## Choice of assay method

**Fig. 2:**
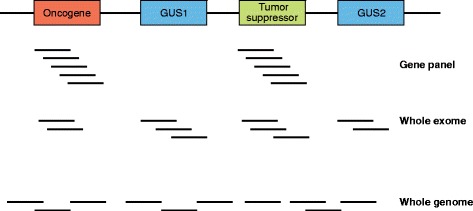
The trade-off between coverage and amount of the genome covered. A hypothetical region of the genome contains an oncogene, a tumor suppressor and two genes of uncertain significance (*GUS*). For visual simplicity, we show ten reads, which will get sequencing depth at genes of interest. Whole-exome sequencing is able to cover each gene with fewer reads, whereas whole-genome sequencing rarely covers a specific base with more than one read. Bear in mind, this figure is vastly understating the relative size of intergenic regions. Realistic sequencing depth goals should be much higher

Before the development of NGS, tumor genotyping was performed only on specific genomic loci that were known to be frequently mutated in cancer, which are known as “hotspots”. These approaches were best suited to recurrent activating mutations in oncogenes, such as in the *KRAS* gene in colon [8] and lung cancer [9]. However, these approaches were insufficient to identify alterations in tumor suppressors (in which an alteration anywhere in the gene may impact its function) or the increasingly complex area of “long tail” hotspot alterations in oncogenes [10]. Thus, current assay options involve approaches that may capture known cancer genes (“gene panels”), whole-exome, whole-genome and/or whole-transcriptome approaches. There are several trade-offs to increasing the portion of the genome that is sequenced. The first is a loss of coverage for the same amount of sequencing (Fig. 2). Coverage, or depth, is defined as the average number of mappable reads at a given locus in your panel. Lower coverage limits the ability to confidently call a variant of low allele fraction to be biologically real and not a technical artifact. A second is that whole-genome and whole-exome sequencing require germline sequencing to enhance the identification of true somatic variants [11], which may uncover incidental clinically relevant inherited disorders (see below).

**Fig. 3:**
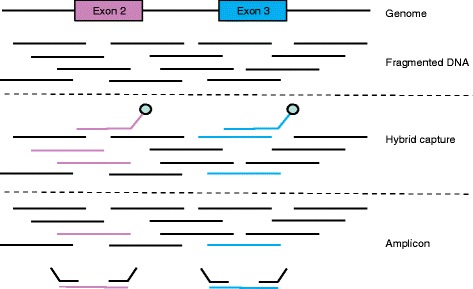
Amplicon-based and hybrid capture sequencing methods. The figure shows a hypothetical gene for which a clinical assay sequences exons 2 and 3. The DNA is sheared either in recovery from being formalin-fixed and paraffin-embedded, or deliberately to allow for sequencing adapter binding. Hybrid capture involves probes that are designed with homology to the gene of interest and bind cDNA. Notice that the fragmented DNA can contain information beyond the boundary of the exon. The probes are biotinylated and unbound DNA is washed away. In amplicon-based sequencing, only probes that contain the complementary sequence for both primers are amplified. Therefore, no information outside of the primers is sequenced

When considering a gene panel, another decision is whether the technology should be based on hybrid capture or amplicon sequencing (Fig. 3). Amplicon sequencing enriches target genes by PCR with a set of primers for exons of selected genes prior to NGS analysis [12]. These protocols have the advantage of less required input DNA and less turnaround time than hybrid capture methods, which is critical for clinical application, but potentially PCR amplification can bias the observed allele fraction. It also pulls information out of a lower percentage of starting material, further increasing the chance of bias in calling copy number variations. The informatics analysis is relatively easy, as any read that does not map to a locus between primers can be disregarded. A downside of this simplicity is that the assay is inherently unable to detect unexpected fusions, because either the 5*'* or 3*'* primer would fail to bind the translocated DNA.

In hybrid capture, relevant DNA sequences are hybridized to probes, which are biotinylated. The biotin is bound to streptavidin beads and then non-bound DNA is washed away [13]. This has the advantage of more reliable detection of copy number changes, although some research groups are using amplicon-based sequencing to detect copy number changes as well [14]. The disadvantages of hybrid capture include a higher required depth of sequencing and a more advanced bioinformatics platform (see below). Hybrid capture does have the ability to detect fusion proteins, as they will be pulled down with the baited DNA. Fusions are still a challenge for hybrid capture, however, because while the fusion protein may be common, the breakpoint itself is found over the full range of an intron [15]. If there is a high suspicion that a sample may contain clinically important fusions, an assay based on cDNA should be considered. These assays will show the fused exon–exon junctions, obviating the need to find the genomic breakpoint [16]. Calling variants and DNA copy number changes can be difficult with both methods (as well as with microarray-based assays) when there is high tumor heterogeneity [17] or low tumor purity [18]. For example, a high copy number gain in a small number of cells may be interpreted as a widespread low copy number gain. Thus, putatively actionable copy number variations are typically validated by fluorescent in situ hybridization in clinical settings.

## Choice of clinical sample

Most specimens that are examined by anatomical pathologists are fixed in formalin (4 % formaldehyde) and embedded in paraffin (FFPE). The formalin introduces crosslinks that can both fragment DNA and cause chemical alterations that may alter sequencing results [19]. Early studies demonstrated that using FFPE specimens in PCR-based sequencing led to more errors than using frozen specimens [20]. Some projects, including The Cancer Genome Atlas (TCGA), required the use of fresh frozen tissue [21]. There has been great progress in altering DNA extraction methods such that FFPE specimens are just as useful for NGS as fresh frozen samples [22]. While there have been some early attempts at using FFPE specimens for other modalities besides DNA sequencing [23, 24], these tests are not yet widely used clinically, and the reliability of FFPE versus frozen samples is less well established. Clinicians should feel comfortable requesting NGS on FFPE samples, and do not necessarily have to handle the specimens differently from other diagnostic samples.

For most cancers, the standard pathological diagnosis will require a direct sample of tissue for biopsy. However, many research groups are exploring the diagnostic and therapeutic utility of “liquid biopsies”. One such source of genetic material for disease monitoring are circulating tumor cells (CTCs). These suffer from a low frequency (approximately 1 cell in 10^6^–10^8^ total circulating cells) and must, therefore, go through an enrichment step. A large number of CTC collection and sequencing protocols have been reported and are being evaluated prospectively [25, 26]. Alternatively, DNA released from apoptotic cells in the tumor can be assayed from the peripheral blood, and is usually referred to as circulating tumor DNA (ctDNA). Progress in utilizing ctDNA was recently reviewed [27], with the authors concluding that this approach shows great promise for the purpose of detecting minimal residual disease [28], or helping to improve diagnosis by looking for mutations specifically associated with a particular disease type [29]. RNA is much less stable than DNA in circulating blood, but RNA species can be preserved in extracellular vesicles and information about tumor recurrence can be gleaned from them as well [30]. However, reproducibility has plagued RNA-based studies, and RNA assays are not yet ready for clinical use [31].

Tumor heterogeneity is both a challenge for liquid biopsies and the reason they can be more useful than tissue biopsies [32]. Initially, mutations with a low allele fraction owing to only being present in a subset of tumor cells may be missed by liquid biopsies, as the low amount of DNA input to the assay is compounded by the low incidence of the mutation. This makes distinguishing low allele fraction mutants from errors that are inherent to high-throughput sequencing very difficult (see below). However, the ability for minimally invasive samples to be sequenced repeatedly over time will allow for faster recognition of known resistance mutations. Sequencing artifacts should be random, but sequences that appear serially can be weighted and followed more closely. It should also be noted that errors in aligning reads to the correct locus will give what appear to be recurrent mutations, so all mutations that are used for serial tracking of tumor burden should be manually reviewed. Overall, there is much promise in sequencing tumor DNA from peripheral blood, but its use is still under investigation and clinicians should rely on other methods for tracking disease progression.

## Clinical NGS data analysis

An additional area of innovation for clinical NGS involves bioinformatic analysis of raw genomic data and rapid clinical interpretation for consideration by the treating clinician. The first step in this process is to assign a genetic location to the read by mapping it on a reference genome [3]. Some percentage of the reads will be “unmappable”, that is, the software cannot assign the sequence to a unique genomic location [33]. An individual genome will have a number of deviations from a reference genome, referred to as single nucleotide variants (SNVs), and/or structural alterations such as insertions, deletions or translocations. Somatic mutation analysis, as is done in cancer, involves a number of additional challenges. There are robust algorithms available for identifying many clinically relevant alterations that occur as point mutations, short insertions or deletions, or copy number aberrations in clinical samples analyzed by NGS [34].

However, as DNA mutations accumulate within a tumor, there can be considerable sequence heterogeneity even within a single primary tumor [17]. It can be very challenging to discern whether a read of a low allele fraction represents a true mutation that exists within a subset of tumor cells or is an artifact that should be discarded. While retrospective research endeavors may not require the identification of all possible clinically actionable alterations in a cohort study, prospective clinical cancer genomics requires increased sensitivity to detect low allelic fraction alterations in impure tumor samples that may impact an individual patient’s care. These issues can be exacerbated by low amount of tumor relative to normal tissue within the sample and mitigated by having more reads, that is, greater coverage. If a detected mutation is the result of a low allele fraction within the sample, the number of reads will rise proportionally with total reads, whereas if it is a technical artifact, the number of reads should be random and can be eliminated from analysis. Estimating tumor percentage from a standard pathology specimen should be helpful for giving an expected allele fraction within the sample, but is prone to very high inter-observer variation [35].

A second challenge is frequent DNA fusions, which represent a significant component of the clinically actionable spectrum of alterations in oncology (for example, *ALK* fusions, *BCR-ABL* fusions). Within NGS data, these events will cause both ends of a read to be mappable, but the whole contiguous sequence is not. This is referred to as a split read, and can be challenging in the presence of a high number of structural rearrangements, such as in cancers with chromothripsis [36]. Notably, since most clinically relevant somatic fusions occur outside of coding regions, whole-exome sequencing assays often miss these variants, and gene panels that are not designed to cover known fusion territories will also be unable to identify these fusion products. Thus, when analyzing a clinical NGS data set, it is critical to understand the analytical limitations of a given assay as represented in the downstream data analysis.

## Clinical interpretation of NGS data

After identification of the set of alterations within a given patient’s tumor, many cases will yield a small set of clinically relevant events as well as a long list of sequencing variants of uncertain significance. An emerging body of interpretation algorithms that automate the clinical relevance of the alterations will enable more rapid clinical interpretation of cancer genomic sequencing data. For instance, one algorithm called PHIAL applies a heuristic method to rank alterations by clinical and biological relevance, followed by intra-sample pathway analysis to determine potentially druggable nodes [22, 37]. As such approaches mature, they will be better equipped to apply tumor-specific “priors” to the genomic data, along with genotype–phenotype therapeutic outcomes data, to enable probabilistic approaches to ranking tumor genomic alterations by clinical relevance.

Furthermore, there are several databases that can be accessed to evaluate the clinical significance of mutations. The first level of analysis is whether the variant you are interested in has been seen before in published reports. A simple concept is that driver mutations are more likely to recur across multiple patients and tumor types. The most common databases used (Table 1) are the Catalog of Somatic Mutations in Man (COSMIC) [38, 39], and TCGA (available for data exploration at multiple sites) [40, 41]. After whittling the mutations down to those that are recurrent, information about therapies and prognostic information can be found at a number of locations. Cancer centers that have created and host these databases include MD Anderson’s Personalized Cancer Therapy [42, 43], Vanderbilt’s My Cancer Genome [44, 45], and the Broad Institute’s TARGET [22, 46]. Each database contains useful information and links to relevant primary literature. Moving forward, there will have to be more steps to improve data sharing, with the creation of a central repository of both sequences and de-identified patient information, but there is no consensus yet for how this process should happen.

**Table 1:** Recommended databases for interpreting somatic mutation results in cancer

Database	Institute	Organized by	Reference
TARGET	BROAD	Gene	[46]
PCT	MD Anderson	Gene	[43]
cBioPortal	MSK	TCGA diseases	[41]
COSMIC	Sanger	Gene	[39]
IntOGen	University Pompeu Fabra	Gene	[73]
My Cancer Genome	Vanderbilt	Disease	[45]
CIViC	Washington University	Variant	[74]
DGIdb	Washington University	Drug/gene interaction	[75]

Each database is listed with hosting institution, website, and the primary search term by which it is organized. *TCGA* The Cancer Genome Atlas

Finally, for NGS technologies that require both somatic and germline testing (for example, whole-exome and whole-genome sequencing), the American College of Medical Genetics has released guidelines outlining which variants should always be reported to patients regardless of whether they are relevant to the presenting illness [47]. Since most of these genes involve non-cancer-related syndromes, there is an increasing need for oncologists to be prepared to receive results that bring up unexpected inherited genetic issues [48]. However, the germline component to clinical oncology NGS testing may have significant diagnostic and therapeutic utility, as demonstrated by the identification of pathogenic germline alterations in men with castration-resistant prostate cancer who respond to PARP inhibition [49], and its role in this arena is evolving rapidly.

## NGS utility

There are three general ways that NGS can aid a clinician. The first is with diagnosis; tumor subtypes that only a few years ago were defined by morphologic criteria are now defined by genetic mutations, either inclusively or exclusively. For example, 15/15 patients in a study looking at fibrolamellar hepatocellular carcinoma had an in-frame fusion between *DNAJB1* and *PRKACA* [50]. The second is finding an appropriate “targeted therapy”, as an increasing number of therapies have indications based on DNA sequencing results (Table 2). Patients who lack the mutation targeted by a drug will not only fail to benefit, but can actually be harmed by inappropriate targeted therapies [51]. The third point at which clinicians stand to benefit from NGS is when a patient stops responding to a targeted therapy with known resistance mutations. In some instances, the resistance mutation may be limited to one or a few loci. For example, resistance to EGFR targeted therapies in cancer very frequently involves a single point mutation, and can possibly be overcome by merely switching to a different agent [52]. However, glioblastoma can become resistant to EGFR targeted therapies via a complicated epigenetic regulation [53]. NGS allows a more complete overview of tumor dynamics, and is more likely to shed light on idiopathic resistance mechanisms than a single gene assay.

**Table 2:** FDA-approved drugs with a companion diagnostic

Drug	Disease	DNA mutation	Action
Imatinib, Dasatinib, Nilotinib, Bosutinib	Chronic myelogenous leukemia	*BCR-ABL1* fusion	Indication for therapy
Ponatinib	Chronic myelogenous leukemia	*BCR-ABL1* fusion	Only indicated for T315I mutations
		T315I resistance mutation	
Erlotinib, Afatinib	Lung adenocarcinoma	*EGFR*	Indication for therapy
		Exon 19 deletions	
		L858R	
Vemurafenib, Dabrafenib	Melanoma	*BRAF* V600E	Indication for therapy
Tramatenib	Melanoma	*BRAF* V600E/K	Indication for therapy
Crizotinib	Lung cancer	*ALK* gene fusions	Indication for therapy
Cetuximab	Colon cancer	*KRAS* codon 12, 13	Contraindication to therapy
Olaparib	Ovarian cancer	*BRCA1* and *BRCA2* mutations	Indication for therapy

Each drug has a specific genomic result that is part of its indication for use. *FDA* Food and Drug Administration

**Fig. 4:**
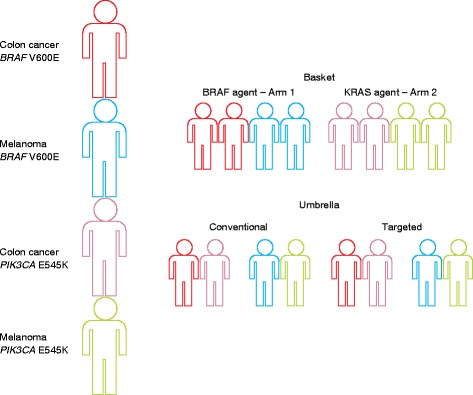
An illustration of new clinical trial designs. Basket and umbrella trials both incorporate genomic data into the basic construction of the trial. Basket trials are designed around specific mutations, regardless of the primary tumor site. Umbrella trials are first separated by primary tumor site and then split into conventional therapy and precision medicine arms

If a patient has failed conventional therapy, NGS can be immensely helpful for identifying and enrolling them into an appropriate clinical trial. There are two types of clinical trial structure that require patients to have their tumors’ genetic makeup well defined by NGS (Fig. 4). In an umbrella trial, patients with a type of morphologically defined cancer are assigned to a treatment arm on the basis of the genetic mutations detected in their tumor. Umbrella trials comprise many different treatment arms under the umbrella of a single trial. In essence, umbrella trials test whether a “precision” approach leads to better outcomes within a traditional diagnosis (for example, lung adenocarcinoma) than standard of care approaches. In a bucket trial (also called a basket trial), cancers of different types are clustered exclusively by genetic mutation. The US National Cancer Institute has recognized the potential of the NGS followed by targeted therapy approach by setting up the Molecular Analysis for Therapy Choice (MATCH) Program. Biopsies from tumors from as many as 3000 patients will undergo NGS to identify individuals whose tumors have genetic abnormalities that may respond to selected targeted drugs. As many as 1000 patients will then be assigned to one of the phase II trials, with assignment based not on their type of cancer but on the genetic abnormality that is thought to be driving their cancer [54]. The nuances of constructing these types of trials are beyond the scope of this review and have been covered well previously [55].

Efforts are ongoing to determine prognostic biomarkers in clinical oncology. Many false starts have been caused by extrapolating from what is called overfitting, which is building a precise model from a small, non-representative data set. Determining prognosis on the basis of non-druggable mutations from NGS has tended to follow from this tradition. Certain mutations, such as *TP53* [56], portend a poor prognosis in almost all clinical situations. Others, such as *ASXL1*, are only associated with a particular disease [57]. Mutations in *IDH1* and *IDH2* indicate a better prognosis in glioma [58], but often show contradictory results in myeloid malignancies [59], although this may change as targeted agents move through clinical trials [60]. Caution should be used when communicating prognostic information to patients.

## Clinical NGS case study

**Fig. 5:**
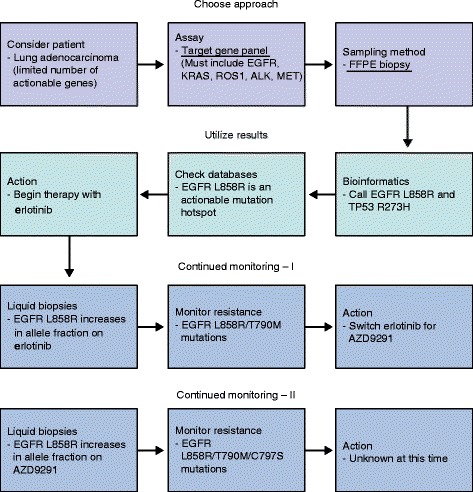
A representative clinical example of how NGS is utilized in recurrent lung adenocarcinoma. The illustrative case from the text has been fitted to the outline in Fig. 1. In a lung adenocarcinoma, there are a number of actionable mutations; this case shows a canonical *EGFR* mutation, treated with erlotinib. There are actually now two levels of resistance that can develop, illustrated in rows 3 and 4. *FFPE* formalin-fixed, paraffin-embedded specimen

As an example that demonstrates the utility of clinical NGS, we look at the fictional scenario of a patient who presents with a newly diagnosed lung adenocarcinoma (Fig. 5). Targeted therapies that affect multiple recurrent alterations in lung adenocarcinoma have been developed, including those that target *EGFR* mutations, MET amplification, and *ALK* or *ROS1* fusions, among others [61]. Thus, a targeted gene panel that encompasses these events would be most commonly applied. DNA can be harvested from the FFPE tumor block obtained from a diagnostic biopsy sample, and targeted NGS sequencing can be used to identify the set of somatic point mutations, short insertions/deletions, copy number alterations, and oncogenic fusion events. In this case, let us say that the resulting inter pretation of the set of variants reveals two mutations: *EGFR* L858R (allelic fraction of 35 %) and *TP53* R273H (allelic fraction of 80 %). All databases highlight that *EGFR* L858R mutations are sensitizing for erlotinib. The *TP53* mutation likely confers a worse prognosis [62], but management does not change as a result. The patient can be followed by both radiology and/or ctDNA assays, with the L858R mutation as a marker of tumor DNA [29]. The patient has a good initial response but develops a recurrence after 6 months. Repeat biopsy and NGS testing is obtained, which reveals the L858R mutation with a 35 % allele fraction and a second *EGFR* T790M mutation with a 12 % allele fraction. From this it would be possible to infer that the second mutation in *EGFR* is derived from a resistant subclone that has emerged as a result of therapy, as indicated by the lower allelic fraction compared with the original *EGFR* mutation. The databases show that this is a common resistance mutation for erlotinib, but can be targeted by newer agents [52, 63]. The patient should continue to be followed, because these newer agents can also trigger the development of additional resistance mutations [52, 64, 65] in *EGFR* or other genes (thereby highlighting the need for broader testing using NGS beyond limited gene testing to ensure identification of the resistance alteration).

## Future directions

While much information can be gleaned from a tumor DNA sequence, we must be mindful that DNA itself is rather inert. Better information about the functionality of a cancer can be obtained by integrating information from different modalities. RNA sequencing could give information about the relative expression of a mutated gene. Approaches in mass spectrometry are giving a clearer picture of the proteomics of cancer [66]. TCGA data were collected using a number of different modalities, and are available for several tumor types, and while useful information can be gleaned at different levels, tying everything together remains a prodigious challenge [67]. The methods used to predict phenotypes from integrated -omics data have been reviewed recently [68].

Furthermore, immunotherapies are quickly gaining prevalence for cancer therapy, especially for use in melanoma [69]. NGS sequencing could become very important for predicting responses to immunotherapy. Neoantigens — that is, antigens that are created by somatic mutations — are correlated with the overall rate of somatic mutation and clinical response [70]. Immune response is mediated by T-cell recognition of these neoantigens [71]. Exome sequencing can be paired with mass spectrometry to determine which neoantigens are successfully presented by the major histocompatibility complex (MHC) [72].

## Conclusion

NGS is inextricably intertwined with the realization of precision medicine in oncology. While it is unlikely to obviate traditional pathologic diagnosis in its current state, it allows a more complete picture of cancer etiology than can be seen with any other modality. However, precision cancer medicine and large-scale NGS testing will require novel approaches towards ensuring evidence-based medicine. Treating each genetic abnormality as an independent variable when hundreds or thousands are queried in every patient will require new trial designs and statistical methods to ensure the utility of these approaches. Broadly, clinicians and translational researchers will need to continue to engage in direct dialog, both within and across institutions, to advance the integration of genomic information and clinical phenotypes, and enable precision cancer medicine through NGS approaches.

## Abbreviations

CTC: Circulating tumor cell
ctDNA: circulating tumor DNA
FDA: Food and Drug Administration
FFPE: Formalin-fixed, paraffin-embedded
MATCH: Molecular Analysis for Therapy Choice
MHC: Major histocompatibility complex
NGS: Next-generation sequencing
SNV: Single nucleotide variant
TCGA: The Cancer Genome Atlas
